# Long noncoding RNA uc.345 promotes tumorigenesis of pancreatic cancer by upregulation of hnRNPL expression

**DOI:** 10.18632/oncotarget.12253

**Published:** 2016-09-26

**Authors:** Chao Liu, Jiamin Wang, Xiaoyuan Yuan, Wenli Qian, Bosen Zhang, Minmin Shi, Junjie Xie, Baiyong Shen, Hong Xu, Zhaoyuan Hou, Hao Chen

**Affiliations:** ^1^ Department of Surgery, Ruijin Hospital, Shanghai Jiaotong University School of Medicine, Shanghai, China; ^2^ Institute of Digestive Surgery, Ruijin Hospital, Shanghai Jiaotong University School of Medicine, Shanghai, China; ^3^ Hongqiao Institute of Medicine, Shanghai Tongren Hospital/Faculty of Basic Medicine, Shanghai Jiaotong University School of Medicine, Shanghai, China; ^4^ Department of Biochemistry and Molecular Cell Biology, Shanghai Key Laboratory for Tumor Microenvironment and Inflammation, Shanghai Jiaotong University School of Medicine, Shanghai, China; ^5^ Key Laboratory of Cell Differentiation and Apoptosis of Chinese Minister of Education, Shanghai Jiao Tong University School of Medicine, Shanghai, China

**Keywords:** pancreatic cancer, lncRNA, uc.345, cancer stem cells, hnRNPL

## Abstract

Increasing evidence points to an important functional or regulatory role of long noncoding RNA in cellular processes as well as cancer diseases resulted from the aberrant lncRNA expression. LncRNA could participate in the cancer progression and develop a significant role through the interaction with proteins. In the present study, we report a lncRNA termed uc.345 that is up-regulated in tumor tissues, compared to the corresponding noncancerous tissues. We found that a higher uc.345 expression level was more frequently observed in tissues with increased depth of invasion and advanced TNM tumor node metastasis T stage. Moreover, uc.345 could be used as an independent risk factor for the overall survival (OS) of the pancreatic cancer patients. By employing soft agar assays and tumor xenograft models, we showed that uc.345 could accelerate tumor growth. Further, we discovered that uc.345 could upregulate the hnRNPL expression and that inhibition of (hnRNPL) dampens the tumorigenesis capability of uc.345. Collectively, these results demonstrate that uc.345 functions as an oncogenic lncRNA that promotes tumor progression and serves as a poor predictor for pancreatic cancer patients' overall survival.

## INTRODUCTION

Pancreatic cancer (PC) is one of the deadliest human malignancies, with a 5-year survival rate of only 5% and a median survival of less than 6 months [1]. The high mortality and poor prognosis of PC is mainly due to its difficulty of making an early diagnosis as well as to its extremely aggressive malignant behavior. About 15–20% of PCs are resectable, and even with surgery the average 5-years survival rate is only 3–5% [2]. Therefore, the searching for novel and biologically relevant genes with prognostic and therapeutic significance in PC are urgently needed.

Accumulating evidence has revealed that long noncoding RNAs (LncRNAs) play essential roles in a wide range of biological processes [3, 4], as well as in the progression of a variety of human diseases, including cancers [5–8]. LncRNAs represent a subgroup of noncoding RNAs that are longer than 200 nucleotides without protein-coding capacity. Recent studies identified several hundred LncRNAs with a size > 200 bp that show a remarkable conservation with 100% identity across the human, mouse, and rat genomes [9]. These highly conserved LncRNAs have been named ultraconserved RNAs (ucRNAs). These ucRNAs in the human genome are most often located either overlapping exons in genes involved in RNA processing or in introns or nearby genes involved in the regulation of transcription and development. So far there are 481 ucRNAs with length arrange from 200 to 779 bp, named as uc.1 to uc.481. 53% of the ucRNAs are classified as nonexonic (“N”, without evidence of encoding protein), while 23% have been designated as exonic (“E”, that overlap mRNA of known protein coding genes), and 24% as possibly exonic (“P”, with inconclusive evidence of overlap with protein coding genes). Their wide distribution in the genome and lack of natural variation in the human population indicate that these ultraconserved regions have important biological functions [10–13]. A recent study reported that ucRNAs exhibit distinct profiles in various cancers and aberrant expression of specific ucRNA is linked with leukemia and carcinoma. They found uc.73 could modulate apoptosis and cell proliferation in colon cancer cells [14]. Moreover, a correlation between some ucRNAs and clinical prognostic factors, such as MYC amplification, has been observed in neuroblastoma [15]. However, there is no any study on the expression and its biological role of ucRNAs in human pancreatic cancer development.

In the present study, we compared the expression profiles of the ucRNAs in paired PC specimens and adjacent normal tissues and identified that uc.345 is upregulated in PC samples and is correlated with the grade of invasion, TNM stage of PC and poor overall survival. Mechanistic investigations demonstrated that 345 could promote tumor growth via an upregulating the hnRNPL expression.

## RESULTS

### mRNAs and ucRNAs are differentially expressed in human PC specimens

**Figure 1:**
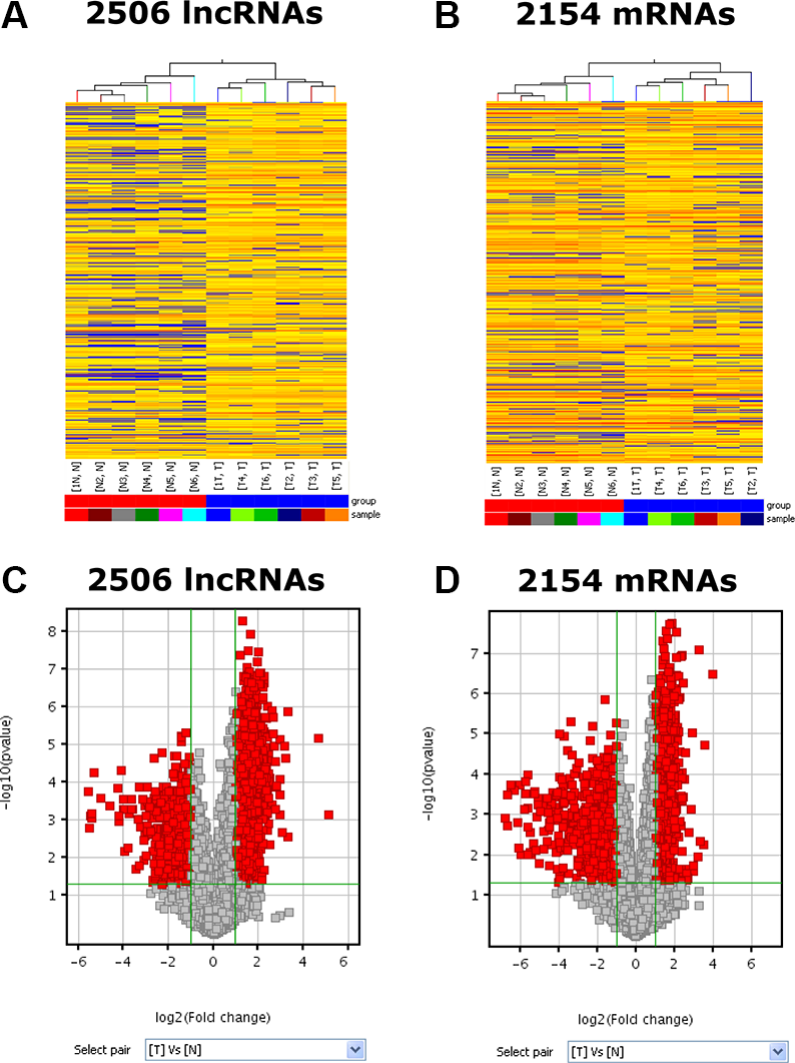
Differentially expressed RNA profiles between human PC tissue and paired adjacent non-tumor samples (**A**, **B**) It shows the expression profiles of the lncRNAs and mRNAs in PC tissue and adjacent normal tissue. (**C**, **D**) The red points in the Volcano Plots represent the differentially expressed lncRNAs and mRNAs with statistical significance. The vertical lines correspond to 2.0-fold up and down and the horizontal line represents a *P*-value of 0.05. Data shown was averages from 6 pairs of PC specimens and adjacent non-tumor tissue.

The RNA expression microarray analyses were conducted on 6 pairs of PC specimens and adjacent normal tissue and the data were deposited into NCBI GEO (GSE86436). Hierarchical clustering analyses were performed to show the distinguishable LncRNAs and mRNAs expression patterns. Total of 33,045 LncRNAs and 30,215 coding transcripts were detected by our second-generation LncRNA microarray. By setting the filter for the Fold Change ≥ 2 and the *P* value ≤ 0.05, we found that 1669 LncRNAs expression were significantly upregulated and 837 LncRNAs were downregulated in the PC group compared to normal pancreatic samples (Figure 1A and 1C). Hierarchical clustering analysis showed that there existed systematic variations in the mRNA expression of 2154 genes between PC and the control group (Figure 1B and 1D). In the PC group, 1200 genes were upregulated and 954 genes were downregulated (Fold Change ≥ 2.0, *P*-value ≤ 0.05)

### ucRNAs are aberrantly expressed and uc.345 expression is increased in PC tissues

**Figure 2:**
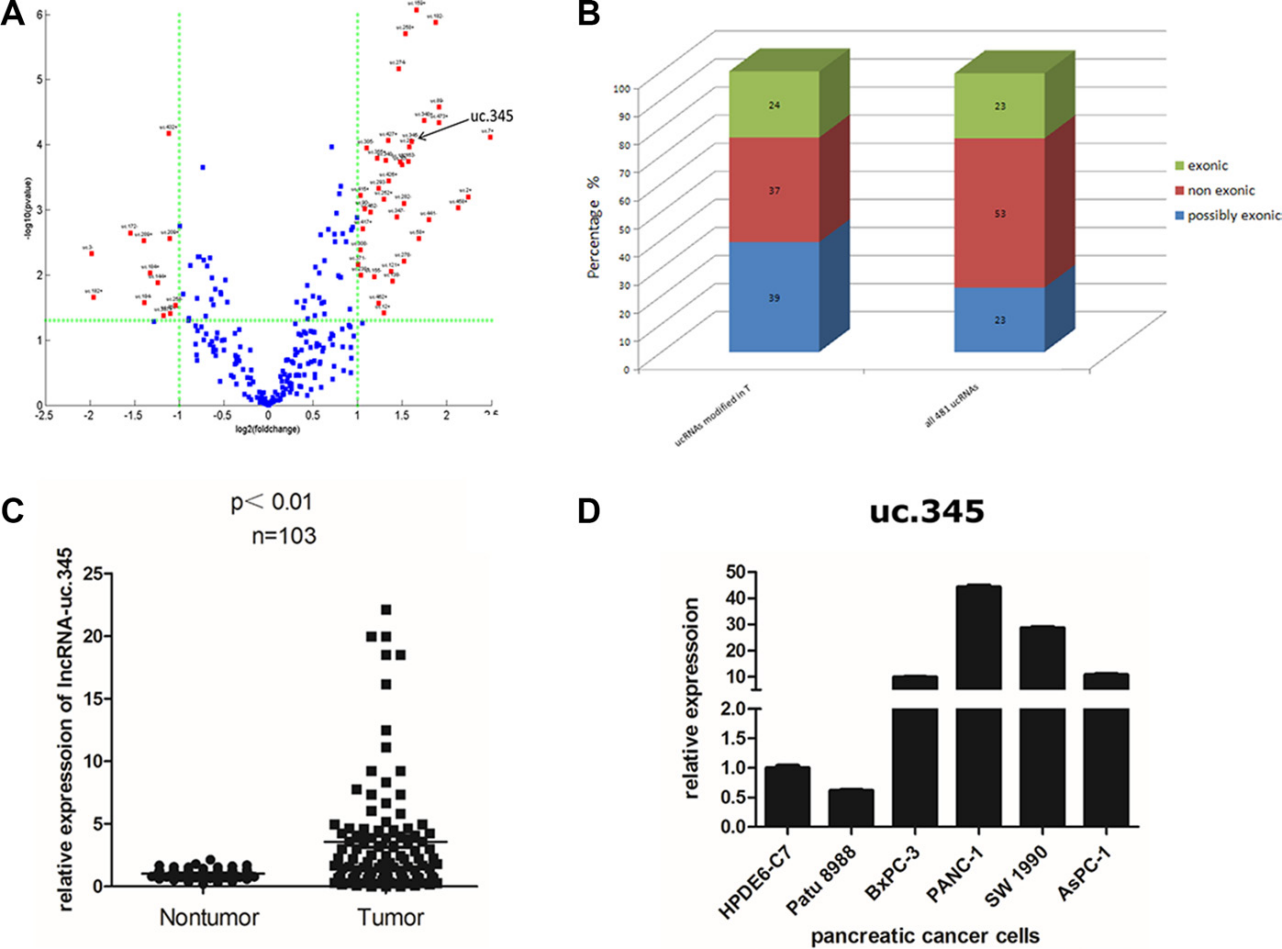
uc.345 is up-regulated in PC tissue (**A**) Genome-wide expression profiling of the ultraconserved LncRNAs (ucRNA) was performed in pancreatic cancer tissues and normal tissues. Total of 261 ucRNAs were aberrantly expressed in PC, with 39 ucRNAs increased and 12 decreased by greater than two fold. The ratio of expression of these ucRNAs in pancreatic cancer tissues to normal tissues was plotted against the *P* value. Selected ucRNAs with a greater than two fold change in expression were annotated. (**B**) The genomic locations of the ucRNA as exonic, nonexonic, or possibly exonic relative to protein-coding genes is depicted for all ucRNAs and for the group of ucRNAs that are aberrantly expressed in pancreatic cancer tissues. (**C**) uc.345 is significantly up-regulated in PC tissues compared to the paired noncancerous tissues. Statistical differences were analyzed using the paired *t* test. Horizontal lines represent the median. (**D**) uc.345 is highly expressed in pancreatic cancer cells except Patu 8988, compared with normal pancreatic cells HPDE6-C7. RNA was extracted from different cell lines and uc.345 expression evaluated by quantitative real-time-PCR. The expression of uc.345 was normalized to that of RNU6B.

The expression arrays identified 261 ucRNAs, representing 54% of all ucRNAs analyzed, that were aberrantly and significantly expressed in PC tissues compared with the adjacent tissues (Fold Change ≥ 2.0, *P*-value ≤ 0.05) (Figure 2A). Of these, 39 ucRNAs were increased in PC tissues, whereas 12 ucRNAs were decreased. Exonic ucRNAs were not selectively enriched in PC tissues, and the proportion of exonic regions in aberrantly expressed ucRNA (24%exonic) was similar to those of all ultraconserved regions (Figure 2B). Notably, we found one ucRNA annotated for ultraconserved element 345(uc.345) was increased in PC tissue. Furthermore, we examined uc.345 expression in PC tissue and adjacent normal tissue from 103 PC patients by qRT-PCR. The results indicated that uc.345 expression was increased in most human PC tissues (68 cases) and 35 cases showed lower expression compared with paired normal adjacent tissues (Figure 2C). Next, we investigated uc.345 expression in several human pancreatic malignant cell lines compared with normal pancreatic epithelial cell line HPDE6-C7. We found a striking increase in uc.345 expression in pancreatic malignant cell lines except Patu 8988 (Figure 2D). Collectively, these data demonstrate that uc.345 expression is increased both in human PC specimens and pancreatic malignant cells.

### uc.345 predicts PC patients with poor survival

**Figure 3:**
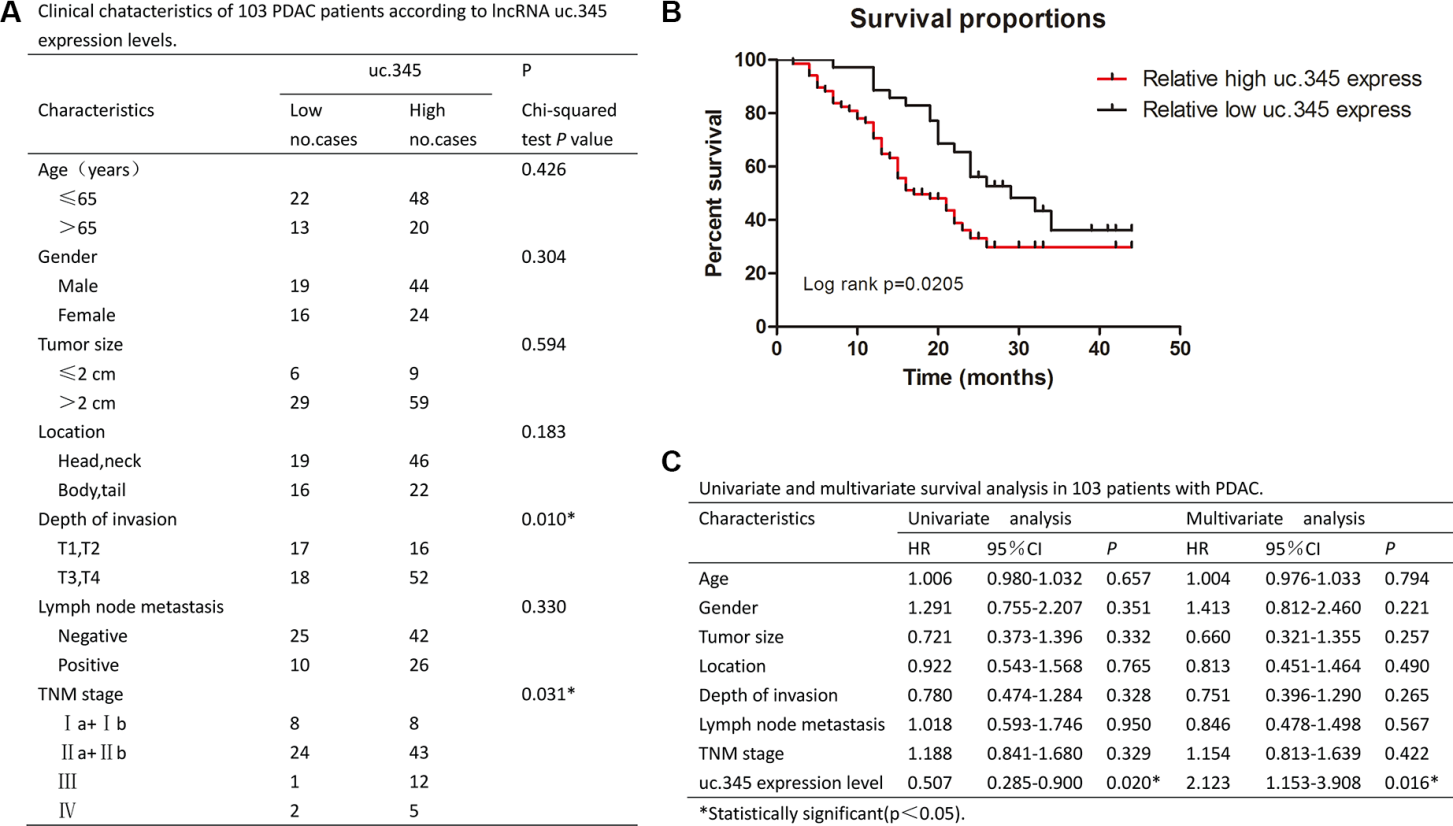
uc.345 is an independent prognostic factor to predict overall survival (**A**) Clinical characteristics of 103 PC patients according to uc.345 expression levels. Relative expression level between tumor and normal tissues was used as the cutoff. For analysis of correlation between uc.345 levels and clinical features, Pearson's chi-square and Fisher's exact tests were used. (**B**) Kaplan-Meier's analyses of correlations between uc.345 expression level and OS of 103 PC patients is shown (long-rank test: *p* = 0.0205). (**C**) Univariate and multivariate survival analysis in 103 patients with PC by employing the method of Cox regression.

The clinicopathological characteristics of the 103 patients were summarized in Figure 3A. The data indicated that there was no significant correlation between uc.345 expression level and age, gender, tumor size, tumor location, or lymphatic metastasis, but it had significant correlation with depth of invasion (*p* < 0.05) and TNM stage(*p* < 0.05). A Kaplan-Meier survival curve was used to compare the low (*n* = 35) and high (*n* = 68) subgroups showing that the survival of patients with lower expression of uc.345 was significantly better than that of higher expression group (*p* < 0.05, Figure 3B).

A univariate analysis revealed that the age, gender, tumor size, location, lymphatic metastasis, depth of invasion, and TNM stage were not significantly correlated with OS, but the uc.345 expression level ([HR]:0.507, 95% CI: 0.285–0.900, *p* < 0.05) was significantly correlated with OS. All the clinicopathological characteristics were further applied for multiple analyses. Cox's multivariate proportional hazards model indicated that only uc.345 expression level ([HR]:2.123, 95% CI: 1.153–3.908, *p* = 0.016) was independent risk factor that affected the OS of PC patients (Figure 3C).

### uc.345 expression is regulated independent of the HOXC4 gene

**Figure 4:**
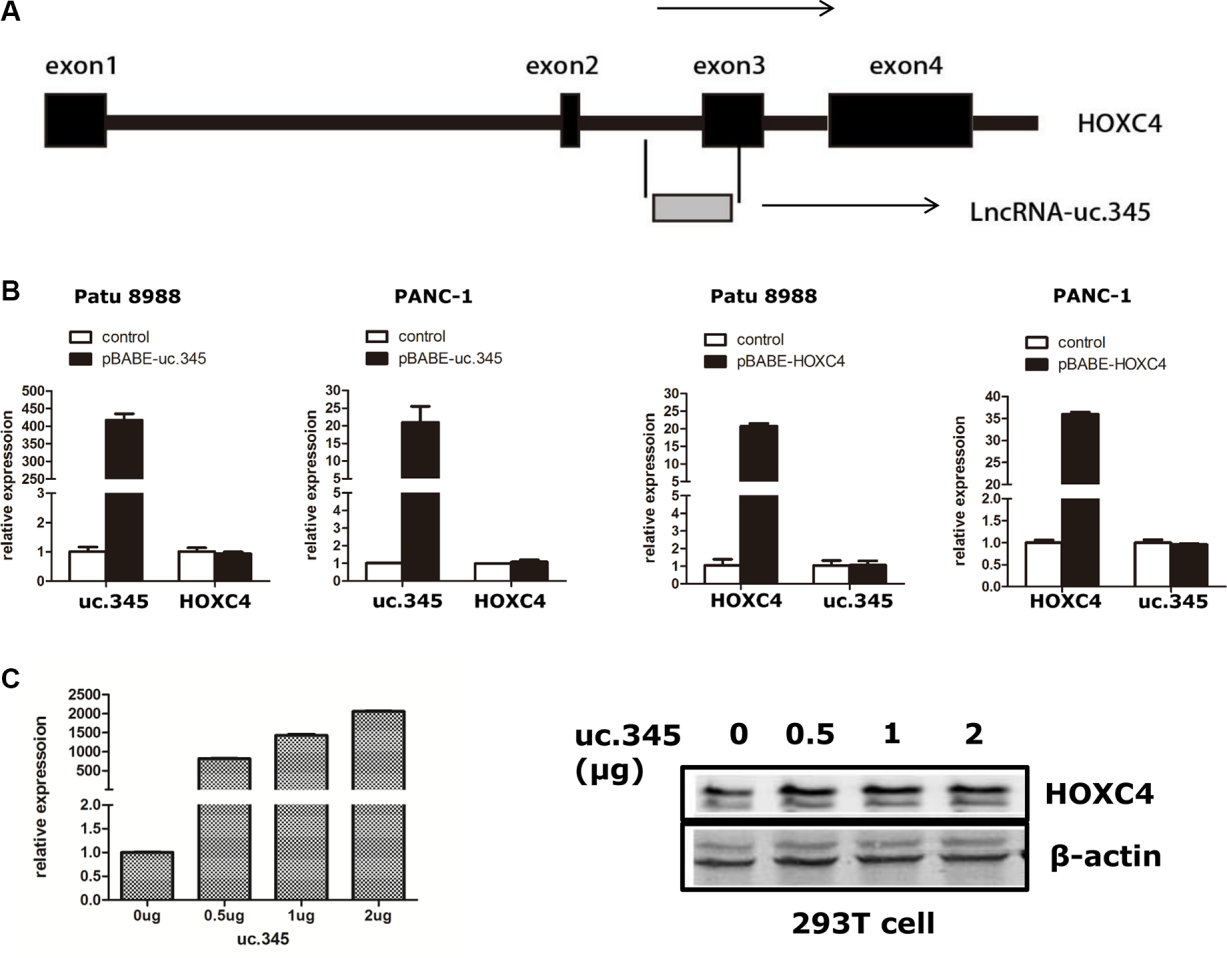
uc.345 and HOXC4 gene are independently regulated (**A**) Schematic representation of the partial exonic location of uc.345 within the HOXC4 gene. The 4 exons of HOXC4 are indicated by dark gray boxes, and uc.345 is indicated by light gray boxes. (**B**) Expression of uc.345 is increased, and HOXC4 mRNA expression is not significantly changed in Patu 8988 and PANC-1 cells. Conversely, increased HOXC4 expression does not change uc.345 expression. Bars represent the mean and SEM of two experiments performed in triplicates. (**C**) The uc.345 expression gradients were detected by qRT-PCR, and with the uc.345 expression increase, there is no effect on HOXC4 protein expression.

uc.345 consists 301 nucleotides that are highly conserved throughout the species. We noticed that the uc.345 is located partly within the exon3 of the HOXC4 gene on chromosome 12 in humans (Figure 4A). To evaluate the potential interrelationships between HOXC4 and uc.345 transcription, we first examined the expression level of HOXC4 and uc.345 by qRT-PCR in Patu 8988 and PANC-1 cell line. HOXC4 transcripts were unchanged in the two cell lines overexpressing uc.345. Conversely, expression of HOXC4 did not affect uc.345 expression in these cell lines (Figure 4B). Furthermore, we transfected uc.345 with increasing amount of plasmids encoding uc.345, and did not observed any apparent effect on HOXC4 protein expression (Figure 4C). Taken together, these data demonstrate that the transcription of uc.345 and HOXC4 gene has no regulative relationship.

### Overexpression of uc.345 promotes pancreatic tumor growth

**Figure 5:**
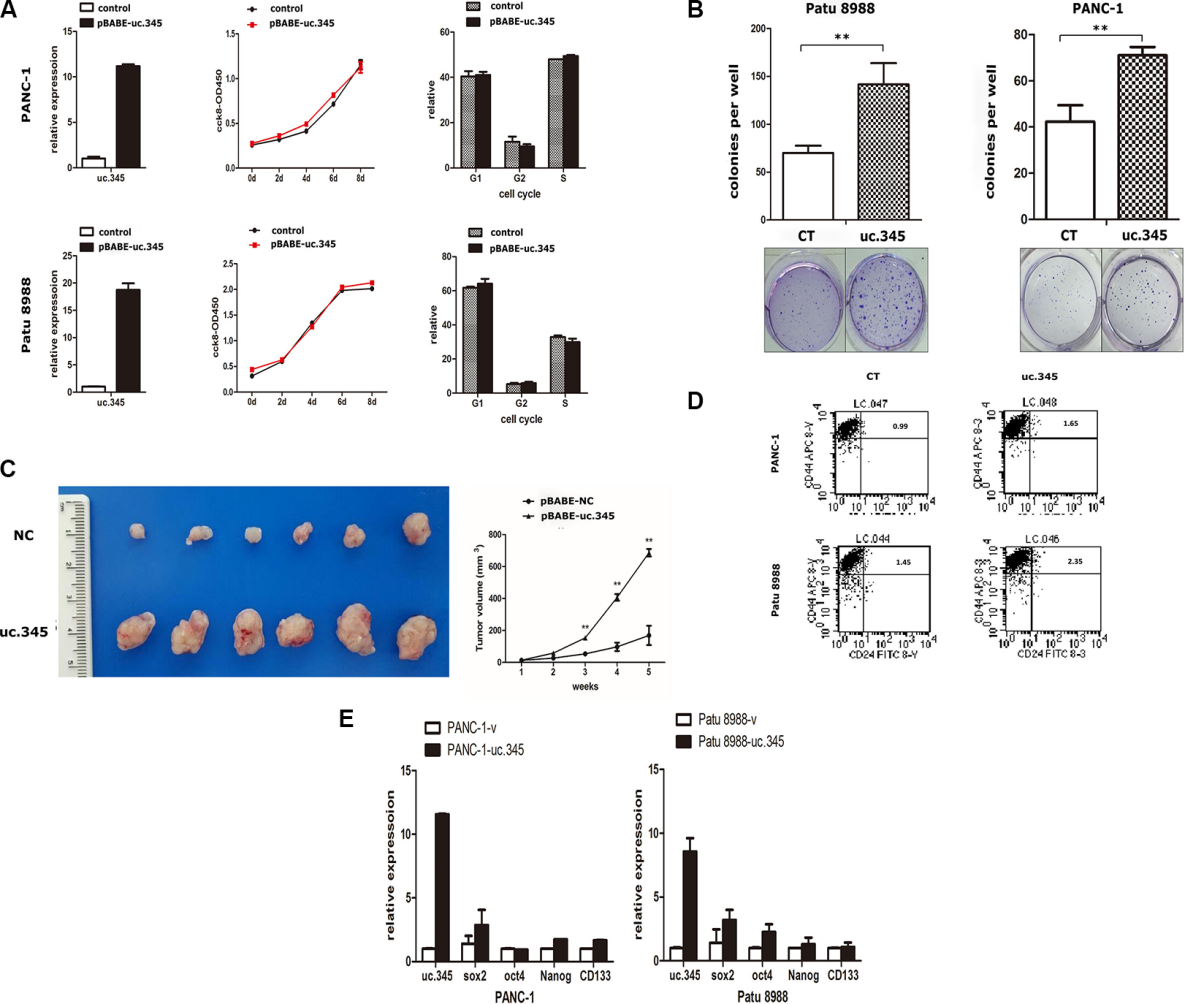
Overexpression of uc.345 promotes tumorigenesis of pancreatic cancer cells *in vitro* and *in vivo* (**A**) PANC-1 and Patu 8988 cells are stably overexpressed uc.345. Growth curves were determined by CCK8 assays and analysis of cell-cycle distribution was performed by flow cytometry. (**B**) Soft agar colony-formation assays were performed to detect the effect of uc.345 on the anchorage-independent growth of PANC-1 and Patu 8988 cells. Colonies were counted after 4 wk. (**C**) Left: Representative images of xenograft tumors after the subcutaneous injection of PANC-1 cell stably overexpression of uc.345 or negative control. Right: Tumor volumes. *P* < 0.01. (**D**) The effect of uc.345 on cancer stem cells in PANC-1 and Patu8988 cells was determined by measuring the percentage of CD24+CD44+ positive cells using flow cytometry. (**E**) qRT-PCR assays were performed to detect some pluripotency-related transcriptional factors such as Oct4, Sox2, Nanog, CD133 markers expression when uc.345 are stably overexpressed in PANC-1 and Patu 8988 cells.

To explore the potential biological function of uc.345 in pancreatic cancer development, we established cell lines stably expressing uc.345 via a lentiviral infection in PANC-1 and Patu 8988 cells respectively. uc.345 expression was markedly increased examined by qRT-PCR (Figure 5A, left). First, we examined the cell proliferation in stably overexpressing uc.345 cells, compared with control pBABE-vector cells. Growth curves determined by CCK8 assays showed that there was no significant change resulted from uc.345 overexpression in both PANC-1 and Patu 8988 cells (Figure 5A, middle). Moreover, we assessed its effect on cell cycle in these two cell lines performed by flow cytometry. The analysis of cell cycle distribution revealed that uc.345 did not change the percentage of G1, G2, S phases, indicating that uc.345 upregulation had no apparent effect on pancreatic cancer cell cycle (Figure 5A, right). Remarkably, colony-formation assays revealed that uc.345 overexpression significantly promoted anchorage-independent growth of PANC-1 and Patu 8988 cells, as indicated by the formation of more and bigger colonies in soft agar (Figure 5B). To further verify these observations, we injected nude mice subcutaneously with PANC-1 cells stably overexpressing uc.345 or vector control. Consistently, tumors formed by uc.345-upregulated cells grew much larger than those formed by control cells (Figure 5C, left). The tumor volume from the uc.345 overexpression group was larger significantly when compared with the control group (Figure 5C, right). These *in vivo* and *in vitro* studies demonstrate that uc.345 has functional roles in regulating PC cell tumorigenesis and tumor growth without effect on cell proliferation.

To explore the potential mechanisms by which uc.345 promotes tumorigenesis, we assessed its effect on caner stem cells (CSCs) in PANC-1 and Patu 8988 cells. In general, a CD44+/CD24+ phenotype has stem-cell properties in pancreatic cancer cells [16–18]. So we detected the population of CD44+/CD24+ cells in uc.345 overexpression groups and the control groups. The results showed that the ratio of CD44+/CD24+ cells in uc.345 overexpression group was significantly increased to 1.65% compared to 0.99% from the control group in PANC1 cells. Similarly, In Patu 8988 cells the ratio of CD44+/CD24+ cells in uc.345 overexpression group was 2.35% compared to 1.45% of the control group (Figure 5D). Moreover, pluripotency-related transcriptional factors such as Oct4, Sox2, Nanog, CD133 were markedly increased by overexpression of uc.345 verified by qRT-PCR (Figure 5E). Taken together, these data suggest that uc.345 promotes tumorigenicity of pancreatic cancer cells by increasing the amount of CSCs.

### hnRNPL is required for uc.345 to promote tumor growth of pancreatic cancer cells

**Figure 6:**
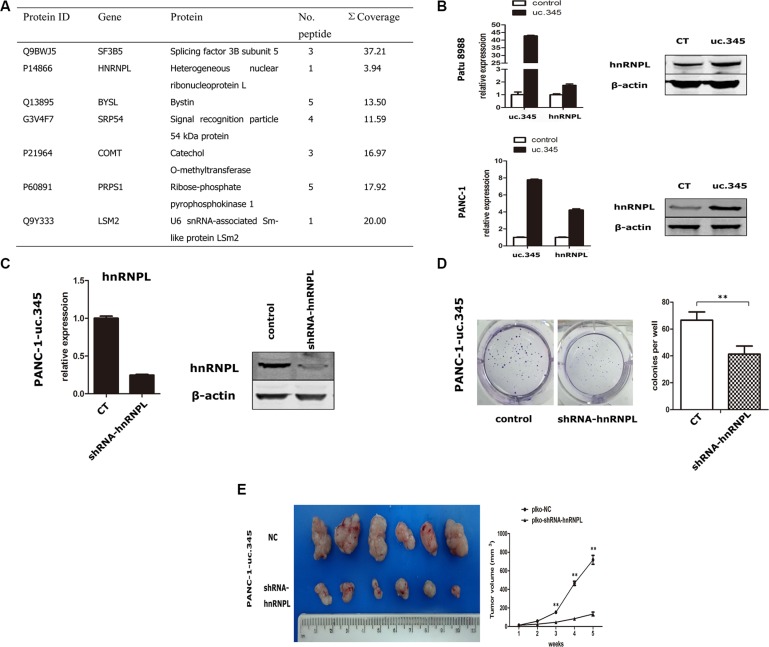
uc.345 promotes PC cells tumorigenecity depending on hnRNPL (**A**) Partial list of the highly and specifically expressed proteins in uc.345 overexpression cells identified by LC-MS/MS. (**B**) mRNA and protein levels of hnRNPL were upregulated in PANC-1 and Patu 8988 cells which stably overexpressed uc.345. The expressions of uc.345 and hnRNPL were normalized to that of RNU6B and β-actin respectively. (**C**) hnRNPL expression was depleted using shRNA, confirmed by qRT-PCR and western blot assays. (**D**) Knockdown of hnRNPL resulted in a dramatic derease in colony-formation in uc.345 overexpression cells by using Soft agar colony-formation assay. (**E**) Left: Representative images of xenograft tumors after the subcutaneous injection of PANC-1 cell stably overexpression of uc.345 with hnRNPL knockdown or negative control. Right: Tumor volumes. *P* < 0.01.

To further determine the potential mechanisms underlying tumorigenesis promotion of uc.345, we collected and analyzed the differentially expressed proteins in Patu 8988 and PANC-1 cells in which uc.345 was overexpressed, compared with control cells by using the LC-MS/MS mass spectrometry. Total of 33 proteins showed high expression in uc.345 overexpressing Patu 8988 and PANC-1 cells and we further picked up 6 most abundant target proteins for validation (Figure 6A). hnRNPL has been shown to play important roles in tumorigenesis, which prompted us to examine the role of hnRNPL in uc.345 mediated tumorigenesis. hnRNPL was confirmed to be upregulated at transcriptional and translational levels when uc.345 was overexpressed (Figure 6B). In order to determine the role of hnRNPL in tumor progression, we designed shRNA to specifically deplete hnRNPL expression PANC-1 cells (Figure 6C). Next, we examined the capability of tumor formation and anchorage-independent growth after knocking-down hnRNPL in uc.345 overexpressing PANC-1 cells. Indeed, the number of colonies formed was dramatically decreased in PANC-1-uc.345-shhnRNPL cells, compared with control (Figure 6D). Further, PANC-1-overexpressing uc.345 cells and cells of PANC-1-overexpressing uc.345 with knocking-down hnRNPL were injected subcutaneously in mice, and showed that depletion of hnRNPL in PANC-1-overexpressing uc.345 cells resulted in smaller tumors (Figure 6E). These data collectively indicate that upregulation of hnRNPL is required for uc.345 to promote pancreatic tumor growth.

## DISCUSSION

Although transcribed ucRNAs were described several years ago [19–21], the potential physiological roles of these ucRNAs in cells still remained elusive. Here, we revealed that a number of the ucRNAs that are aberrantly expressed in human PC, and particularly, we confirmed that uc.345 is highly expressed in PC. The level of uc.345 is significantly associated with cancer phenotypes, including depth of tumor invasion, clinical TNM stage, and as an independent risk factor to predict overall survival. These findings expand the number of valuable biomarkers for pancreatic cancer diagnosis and prognosis.

Biologic investigations demonstrated that uc.345 can promote pancreatic cancer development evidenced by enhancing colony formation in the soft agar assays, and accelerating tumor growth in nude mice. However, we did not find that up-regulated uc.345 affects the cell proliferation or cell cycle of pancreatic cancer cells. Interestingly, we did find that overexpression of uc.345 results in increased populations of CD44+/CD24+ cells in PANC-1 (from 0.99% to 1.65%) and Patu 8988 (from 1.45 to 2.35) cells, and consistently, uc.345 could increase pluripotency-related transcriptional factors such as Oct4, Sox2, Nanog, CD133 expression. Although the portions of the increased CSCs by uc.345 is only about 1% or so, we did persistently observe the phenotype of CSC increase when uc345 is overexpressed in multiple pancreatic cancer cell lines. Thus, we think the increased CSCs at least partially contribute to the accelerated tumor growth phenotype by uc345. We definitely want to pursue further to determine the role of uc345 in the regulation of CSC and promotion of tumor growth.

hnRNPL is an RNA binding protein found inside and outside the nucleolus and associates with hnRNAs (such as pre-mRNAs and mRNAs) involved in the formation, packaging, and processing of mRNA [22–25]. Splicing targets of hnRNPL affect diverse processes, including tumorigenesis [26, 27], T-cell activation [28–31], vasculogenesis [32], and signal transduction [33, 34]. Our data demonstrate that uc.345 increases both hnRNPL transcripts and the protein levels and knocking-down hnRNPL with specific shRNA suppresses the tumorigenetic capability of uc.345, indicating hnRNPL is an important downstream target of uc.345 to promote tumorigenesis.

The mechanism by which uc.345 regulates hnRNPL expression remains an interesting question needed to be elucidated further. That LncRNAs regulate gene expression are very complex processes in which multiple mechanisms have been reported: 1) LncRNAs can act as cis-acting factors, or trans-acting factors, or even as enhancers; 2) LncRNAs can regulate gene expression by post-transcriptional modifications. However, we do not know that the molecular mechanism by which uc.345 regulates hnRNPL expression. We definitely want to explore the molecular event that how uc.345 regulates hnRNPL in cells.

## MATERIALS AND METHODS

### Pancreatic tissue collection

We obtained 103 pairs of primary PC and adjacent non-tumor tissues from patients undergoing resection surgery at the Department of General Surgery, Ruijin Hospital in Shanghai from 2011 to 2013. For all cases, two pathologists were in agreement with regard to pathological features and confirmed the PC diagnoses. No local or systemic treatment was conducted in these patients before surgery. Clinical characteristics of all patients enrolled in the study were summarized in Figure 3A. All samples were snap-frozen in liquid nitrogen immediately from resected PC cases and stored at −80°C. The study was approved by the Ethic and Research Committees of Ruijing Hospital, Shanghai Jiaotong University School of Medicine and was conducted in accordance with the Declaration of Helsinki Principles. The procedures for pancreatic cancer resection were described in detail to all patients before admission, and informed consent of patients was obtained.

### LncRNA microarray

Total RNAs were extracted using Trizol reagent from each PC sample, quantified by the NanoDrop ND-1000 and the RNA integrity was assessed by standard denaturing agarose gel electrophoresis. For microarray analysis, Agilent Array platform was employed. The microarray hybridizations were performed based on the manufacturer's standard protocols with minor modifications. Briefly, mRNA was purified from total RNA after removal of rRNA (mRNA-ONLY™ Eukaryotic mRNA Isolation Kit, Epicentre). Then, each sample was amplified and transcribed into fluorescent cRNA along the entire length of the transcripts without 3′ bias utilizing a random priming method. The labeled cRNAs were hybridized onto the Human LncRNA Array v2.0 (8 × 60 K, Arraystar). After having washed the slides, the arrays were scanned by the Agilent Scanner G2505C. Agilent Feature Extraction software (version 11.0.1.1) was used to analyze acquired array images. Quantile normalization and subsequent data processing were performed using the GeneSpring GX v11.5.1 software package (Agilent Technologies). After quantile normalization of the raw data, LncRNAs and mRNAs were chosen for further data analysis. Arraystar Human LncRNA Microarray v2.0 is designed for the global profiling of human LncRNAs and protein-coding transcripts. 33,045 LncRNAs and 30,215 coding transcripts can be detected by our second-generation LncRNA microarray. The LncRNAs are carefully collected from the most authoritative databases such as RefSeq, UCSC Known genes, Ensembl and many related literatures. Differentially expressed LncRNAs and mRNAs with statistical significance were identified through Volcano Plot filtering. The threshold set for up- and down-regulate genes was a fold change ≥ 2.0 and a *P*-value ≤ 0.05. Pathway analysis and GO analysis were applied to determine the roles of these differentially expressed mRNAs played in these biological pathways or GO terms. Finally, Hierarchical Clustering was performed to show the distinguishable LncRNAs and mRNAs expression pattern among samples.

### Cell culture

HEK-293T cells and pancreatic cancer cells PANC-1 and AsPC-1 were obtained from the American Type Culture Collection(Rockville, MD, USA) and HPDE6-C7, Patu 8988, BxPC-3 and SW 1990 cells were purchased from the Institute of Biochemistry and Cell Biology of the Chinese Academy of Sciences(Shanghai, China). All cells were tested and authenticated by DNA typing at the Shanghai Jiao Tong University Analysis Core. The cells were maintained in DMEM or RPMI 1640(Gilbco) supplemented with 10% FBS, 2 mmol/L L-glutamine, and penicillin (50 U/mL)/streptomycin (50 mg/mL) at 37°C under 5% CO_2_ in a humidified chamber.

### Plasmids and transfections

uc.345 cDNA was cloned into pBABE-vector between BamHI and EcoRI sites. pLKO.1-shRNAs targeting hnRNPL were TGCTGTTGACAGTGAGCGA CAGCTTT TGTCAACTACTCTATAGTGAAGCCACG ATGTATAGAGTAGTTGACAAAAGCTGGTGCCTAC TGCCTCGGA. We cultured cells stable uc.345 overexpression by using lentivirus-based delivery method. The viral supernatants were generated in HEK-293T cells, and were infected into PANC-1 and Patu 8988 cells. Puromycin was added into the media to generate stable uc.345 overexpression in PANC-1 and Patu 8988 cells. Using the same way to generate stable knockdown of hnRNPL in PANC-1 cells. Transfection of HEK-293T cells was performed using Lipofectamine 2000(Invitrogen) according to the manufacturer's instructions.

### RNA extraction and quantitative RT-PCR assays

Total RNA was isolated with Trizol reagent (Invitrogen) according to the manufacturer's instructions. Total RNA (2 μg) was reverse transcribed in a final volume of 10 μl using random primers under standard conditions for the PrimeScript RT Reagent Kit (Invitrogen). qRT-PCR was performed on the 7500 Fast Real time PCR system (Applied Biosystem) using SYBR Green agent (Applied Biosystem) to determine uc.345 expression levels, following the manufacturer's instructions. Results were normalized to the expression of RNU6B. Primers used for qRT-PCR assay were listed in Supplementary Information. Our qPCR results were analyzed and expressed relative to threshold cycle (CT) values and then converted to fold changes. All qRT-PCR assays were repeated three times.

### Western blot

The methods used for Western blot were previously described [35]. A primary antibody against HOXC4(Proteintech), hnRNPL(Aviva Systems Biology) and a secondary antibody (antirabbit IgG, Santa Cruz Biotechnology)were purchased. Equal protein specimen loading was monitored using an anti-β-actin antibody (Cell Signaling Technology).

### Cell growth counting and cell cycle assays

For cell growth counting assays, multiple cultures of pancreatic cancer cells were plated in 96-well plates at a density of 1.0 × 10^3^ cells per well. Three wells for each cell line in each of three biological repeats of the experiment. Every two days one set of cultures was tested followed by cell counting kit-8 at 450 nm. For cell cycle assays, pancreatic cancer cells (2~5 × 10^5^)stable overexpression uc.345 or negative control (N.C) were plated in 6-well plates. After 48 h incubation, cells washed with phosphate-buffered saline (PBS), and fixed in 75% ethanol at 4°C overnight. RNA was removed from the preparations by incubating with RNaseA (Sigma-Aldrich) at 37°C for 30 min. Cells were then stained with propidium iodide (PI) solution (Sigma-Aldrich) for 30 min at room temperature and analyzed on FACS Aria I flow cytometer (BD Biosciences).

### Colony formation assay

Colony-forming efficiency was determined by using a double-layer soft agar method. A total of 0.5~ 1.0 × 10^4^ cells were plated in 0.35% agar over a layer of 0.5% agar containing DMEM and 10% FBS in 6-well plates. After 2 or 3 weeks of growth, the cells were washed in PBS 3 times and dyed with methylrosanilnium chloride solution at a 0.01% concentration for counting.

### Flow cytometric analysis

Dissociated cells were counted and transferred to a 5-ml tube, washed twice with PBS containing 2% heat-inactivated FBS, and resuspended in PBS with 2% FBS at a concentration of 10^7^ cells per 100 μl. Antibodies at the appropriate dilution were added to the cells, and the mixture was incubated for 30 min on ice. Then, the sample was washed twice with PBS containing 2% FBS. The antibodies were anti-CD44 allophycocyanin (APC) (eBioscience, San Diego, CA, USA), anti-CD24 phycoerythrin (FITC) (eBioscience, San Diego, CA, USA). Flow cytometry analysis was performed by using a MACSQuant analyzer (miltenyi biotec, Gladbach, Germany), and results were analyzed with FlowJo software (TreeStar, OR, USA).

### LC-MS/MS

All samples were homogenized in lysis buffer (4% SDS, 1 mM DTT, 150 mM Tris-HCl pH 8.0, protease inhibitor). After 3 min incubation in boiling water, the homogenate was sonicated on ice. The crude extract was then incubated in boiling water again and clarified by centrifugation at 16000 × g at 25°C for 10 min. Protein content was determined with the BCA protein assay reagent (Thermo Scientific). Experiments were performed on a Q Exactive mass spectrometer that was coupled to Easy nLC (Thermo Fisher Scientific). MS/MS spectra were searched using MASCOT engine (Matrix Science, London, UK; version 2.2) against uniprot human (136615 sequences, downloaded on 20140802). For protein identification, the following options were used. Peptide mass tolerance = 20 ppm, MS/MS tolerance = 0.1 Da, Enzyme = Trypsin, Missed cleavage = 2, Fixed modification: Carbamidomethyl (C), Variable modification: Oxidation(M), FDR < 0.01 at peptide and protein level.

### *In vivo* mouse study

All animal experiments were overseen and approved by the Animal Welfare Committee of Shanghai Jiaotong University School of Medicine. Tumor growth ability of uc.345-PANC-1 cells was determined by subcutaneous injection into 5-week-old female BALB/c athymic nude mice (nu/nu, Slac Laboratory Animal Co. Ltd., Shanghai, China). Six mice were used in each group. A total of 3 × 10^6^ cells were injected subcutaneously into the left rear or right rear flank per mouse. Mouse tumor volume was measured every seven days. The width (W) and length (L) of the tumor were measured using a digital caliper, and the tumor volumes were calculated using the formula V = 1/2 (L × W2), where L is the length (longest dimension) and W is the width (shortest dimension). Tumor growth was followed for 1 month after tumor cell injection.

### Statistical analysis

The results were represented as the mean ± standard deviation (SD). The statistical significance of differences was examined by Student's *t* test, Pearson's chi-square or Fisher's exact test in two groups. The survival calculations were illustrated with Kaplan-Meier curve, and univariate and multivariate analyses were done using the log-rank test or the Cox proportional hazards regression model. Significant differences among groups were calculated at *p* < 0.05. All data were analyzed with SPSS statistical software (SPSS, Inc. Chicago, IL, USA).

## SUPPLEMENTARY MATERIALS


