# Supplementary material for: Long noncoding RNA uc.345 promotes tumorigenesis of pancreatic cancer by upregulation of hnRNPL expression
Source: Oncotarget. 2016 Sep 26;7(44):71556–66. doi: 10.18632/oncotarget.12253 (PMC5342101; doi:10.18632/oncotarget.12253)
Supplement: Supplementary file 1 [file oncotarget-07-71556-s001.pdf]

Long noncoding RNA uc.345 promotes tumorigenesis of pancreatic cancer by upregulation of hnRNPL expression

Supplementary Materials

qRT-PCR primer

RNU6B primer F
5′-GCTTCGGCAGCACATATACTAAAAT-3′
primer R
5′-CGCTTCACGAATTTGCGTGTCAT-3′
uc.345 primer F
5′- AAGTAACTTTACAGGGTCGCT -3’
primer R
5′- TTCTGCTCCATAAAGCCCTCC -3′
HOXC4 primer F
5′- CTACCTGACCCGAAGGAGAA -3′
primer R
5′- TGACCTCACTTTGGTGTTGG -3’
hnRNPL primer F
5′- AGATCACCCCGCAGAATATG -3′
primer R
5′- CAAGCCATAGACCATGAGCA -3′

PCR primer

uc345 PCR primer F
5′- CGCGGATCCGGAGTCACATGGTGAAAG -3′
PCR primer R
5′- ATAGAATTCCGCTCAGGGTAGCTAGGG -3′
HOXC4 PCR primer F
5′- CGCGGATCCATGATCATGAGCTCGTAT -3′
PCR primer R
5′- ATAGAATTCTTATAACCTGGTAATGTC -3′

uc.345 sequence

GGAGTCACATGGTGAAAGTAACTTTACAGGGTCG
CTAGCTAGTAGGAGGGCTTTATGGAGCAGAAAAA
CGACAAAGCGAGAAAAATTATTTTCCACTCCAGA
AATTAATGATCATGAGCTCGTATTTGATGGACTCT
AACTACATCGATCCGAAATTTCCTCCATGCGAAG
AATATTCGCAAAATAGCTACATCCCTGAACACAG
TCCGGAATATTACGGCCGGACCAGGGAATGGGAT
TCCAGCATCACCACCAGGAGCTGTACCCACCACC
GCCTCCGCGCCCTAGCTACCCTGAGCG.
